# Current Perspectives on Contemporary Rheumatic Mitral Valve Repair

**DOI:** 10.1177/15569845211032942

**Published:** 2021-09-03

**Authors:** Chaninda Dejsupa, Taweesak Chotivatanapong, Massimo Caputo, Hunaid A. Vohra

**Affiliations:** 1 1980 Department of Cardiac Surgery/Cardiovascular Sciences, University of Bristol, UK; 2 59070 Department of Cardiothoracic Surgery, Central Chest Institute of Thailand, Nonthabhuri, Thailand

**Keywords:** rheumatic heart disease, mitral valve, valve repair, rheumatic valve

## Abstract

The surgical management of rheumatic mitral valve disease remains a challenge for cardiac surgeons. Durability of mitral valve repair (MVr) is likely compromised not simply due to high technical demand, but surgeon reluctance, despite boasting copious advantages over MV replacement. This comprehensive review aims to evoke a deeper understanding of MVr concepts necessary to abate these limitations and shift mindset towards a more holistic approach to repair. Details of commonly utilized techniques in contemporary MVr for rheumatic heart disease will be discussed. Of importance, the reparative procedures will be mapped to an in-depth physiological exploration of the mitral complex-dynamism and rheumatic interplay. This is further emphasized by outlining the current “aggressive” resection strategy in contemporary rheumatic MVr.

**Central Message**

Durability of rheumatic mitral valve repair is likely compromised by a surgeon’s reluctance to attempt repair of complex valves. A deeper understanding of the mitral complex-dynamism and underlying pathophysiology can help abate this.

## Introduction

The superiority of valve repair over replacement has been well-established in degenerative mitral valve (MV) disease, whereas its role in rheumatic heart disease (RHD) has remained controversial. Predominantly due to its notoriously complicated pathology, the repaired rheumatic MV is often believed to have inferior durability due to the ongoing inflammatory process and resultant risk of failure and reoperation.^1-3^ Furthermore, the complexity of RHD frequently requires numerous repair techniques to be used concurrently, demanding the proficiency of high-volume specialist surgeons.^4-6^

Nevertheless, numerous advantages favor MV repair (MVr) over replacement (MVR) in RHD, including lower mortality rates, preservation of ventricular function, elimination of complications related to anticoagulation, and lower risk of endocarditis and thromboembolism.^6-9^ Although data regarding durability of rheumatic MVr remain contentious, there has clearly been significant improvement in recent years. Reports on contemporary rheumatic MVr have highlighted feasibility and excellent outcomes;^10-14^ an overview of several rheumatic MVr series is shown in the Supplemental Table. Chauvaud et al.^12^ reported 29-year results for isolated MVr in RHD, with a 10-year and 20-year actuarial survival of >80% and 20-year freedom from reoperation of 55%. It should be noted the authors excluded patients with other associated valve lesions and coronary artery disease, but these patients were included in the DiBardino et al. series,^1^ which found similar survival rates. Furthermore, not only have comparable 10-year survival and reoperation rates between rheumatic and degenerative MVr been demonstrated, but also a noninferior 10-year freedom from valve failure of 81%, which is an important indicator of durability.^10^ Concerns regarding reproducibility, reliability, and long-term durability are being constantly addressed by standardizing techniques and employing new concepts.^4,10,12^ Thus, despite rheumatic MVr being technically and pathologically challenging, many surgeons favor valve reconstruction to be the preferred primary correction.^11,12,15^

## Shifting Mindset Towards Holistic Approach to Repair

It is of utmost importance to emphasize the most vital benchmark of MVr since Carpentier’s “*French Correction*,”^16^ which is good coaptation. Although this simple aim underlies both rheumatic and nonrheumatic MVr, its implications are extensive. First, adopting the same reconstructive process across etiologies would be flawed; the unique features of rheumatic lesions demand equally unique techniques. Second, the principle should serve to guide the actual repair approach including which techniques suit the intraoperative context, which order should they be performed in, and for what purpose. This necessitates a much deeper physiological understanding of the interconnectivity between the MV complex and dynamics during the cardiac cycle.^11^ Of note, diastolic function tends to be of secondary concern and is often assumed to be corrected once systolic function is addressed. Third, surgeons should attempt to primarily restore the dynamics of the MV in RHD, especially that of leaflet pliability, by preserving any native tissue remaining. Only when the valve is truly past reparable would geometrical reconstruction via MVR provide a good alternative.

The long-standing principle reminds us to tailor techniques more holistically, a matter concerning the surgeon’s mentality more than technical dexterity. Deeper understanding of normal mitral physiology and anatomy specific to RHD aids in this simple mindset shift, lowering a surgeon’s reluctance to perform MVr. Thus, this paper attempts to accomplish this through a detailed exploration of commonly utilized MVr techniques and implications for future strategies.

## Surgical Techniques

### Overview of Aims

In RHD, the mitral leaflets/subvalvular apparatus are plagued by varying degrees of fibrosis and calcification due to the inflammatory process, manifesting clinically as mitral stenosis (MS), mitral regurgitation (MR), or both (mixed). Most commonly, rheumatic lesions include severe leaflet thickening, commissural fusion, and subvalvular apparatus shortening/fusion.^7,11^ The net effect is restriction during systole and obstruction during diastole. It is to be noted that the extent of pathology dictates functional outcome due to increased complexity and higher risk of residual diseased tissue post-repair; another well-known factor in successful MVr is the absence of acute rheumatic valvulitis at surgery.^2,17^ Between restoration of MV geometry or normal mitral dynamics/pliability, the priority lies in the latter. This is significant because the left ventricle (LV) is a single chamber that must accommodate both the inflow and outflow of blood. A harmonious system between the LV and remarkably dynamic MV complex enabling unidirectional blood flow is integral in determining normal fluid dynamics. Rheumatic pathology disrupts this balance, which is highlighted by intraoperative echocardiographic assessment and surgical guidance. Overall, the aim of MVr is to enable full leaflet mobilization by optimizing both diastolic and systolic function in the most efficient and least stressful manner (Table 1).

**Table 1:** Summary of Aims for Mitral Valve Repair in Restoring Diastolic and Systolic Function.

Restoring diastole	Restoring systole
Fully open	No restrictions
No restrictive or obstructive movement	Pliability
Fenestrated channels between chords	Tight seal with good coaptation
Pliability of leaflets and chords	Type 1 (Carpentier) leaflet mobility
	Strengthened annulus via ring

### Surgical Approach

The surgical approach begins with systematic valve analysis and echocardiographic assessment. A modified Guiraudon incision provides excellent exposure, which is crucial in successful MVr.^11^ Next, Type 1 Carpentier^16^ mobility should be reconstructed as best as possible in all leaflet segments to restore good coaptation, utilizing a blend of techniques. Of note, MVr is always stabilized with a prosthetic annular ring. Concomitant tricuspid and/or aortic valve disease is then addressed accordingly. Table 2 provides a concise summary of the main repair techniques utilized for different components of the MV complex affected by rheumatic pathology (Supplemental Video).

**Table 2:** Concise Summary of Main Repair Techniques Utilized for Different Components of the Mitral Valve Complex Affected by Rheumatic Pathology.

Mitral valve component	Rheumatic pathology	Main repair technique(s)
Annulus	Heterogenous deformities	Complete ring annuloplasty (semi-rigid or rigid)
Leaflets	Commissural fusion	Commissurotomy
	Thickened leaflets	Peeling
		Pericardium augmentation
Subvalvular apparatus	Short, thick chords	Resection of any obstructive or restrictive chords
		Chordal transfer (if good native chords)
		Polytetrafluoroethylene neochord replacement
	Fused chords	Fenestration
		Papillotomy

### Leaflets

Leaflet procedures are increasingly used in advanced rheumatic lesions, especially in mixed MV disease. Rheumatic mitral leaflets are often retracted and contain excessive fibrous tissue; several techniques have been used to combat this problem.

#### Commissurotomy

**Fig. 1:**
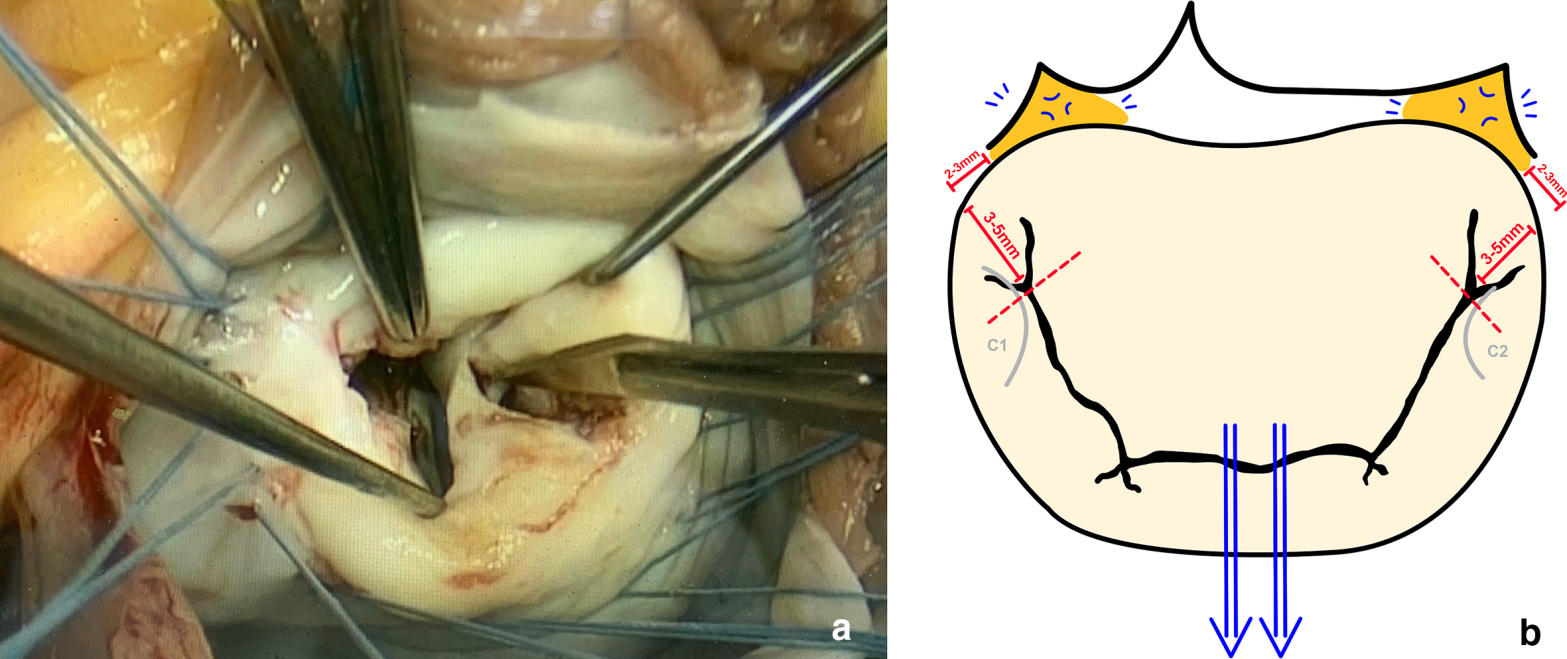
(**a**) Intraoperative antero-lateral commissurotomy being performed, and (**b**) a schematic illustration of how commissurotomy is performed at our institution. The blue arrows depict symmetrical traction, applied by using nerve hooks around the major chordae on the left and right side of the anterior mitral leaflet perpendicular to the inter-trigonal line; this will create areas of dimpling, depicted by the blue markings, subsequently identifying the trigones (orange zones). The red dotted line represents the ideal point up to which commissurotomy should be performed, determined by stopping 3 to 5 mm from the annulus and 2 to 3 mm laterally from the trigone, as represented by the red bars. The commissural leaflets (C1 and C2) are outlined in grey to highlight the importance of preserving the commissural chords.

In subvalvular fusion and leaflet restriction, commissurotomy is frequently performed to better assess leaflet mobility.^1,11,15,18^ In contrast to degenerative valve disease, calcification commonly affects the leaflets, commissures, and subvalvular apparatus in RHD. First described by Carpentier,^16^ commissurotomy is used to correct commissural fusion and is one of the most frequently employed “classic” techniques. In our experience, commissurotomy is performed by applying symmetrical traction with nerve hooks around the major chordae on the left and right side of the anterior mitral valve leaflet (AMVL) perpendicular to the inter-trigonal line; areas of dimpling will identify the trigones. Then, commissurotomy is performed until 2 to 3 mm lateral to the corresponding trigone and 3 to 5 mm from the annulus. Preservation of the commissural chords is key (Fig. 1). In our experience, this method of visualizing the two distances has provided successful clinical outcomes.

Fused commissures in RHD cause restrictive flow and impaired stress distribution on leaflets. Thus, commissurotomy is integral for fully opening the MV during diastole and forming a tight seal during systole. To understand why this is, it is important to remember that the MV is not bileaflet, but a quadri-leaflet structure. The commissural leaflets (C1 and C2) are often given little attention;^19,20^ however, they are more than just additional leaflet tissue. In a healthy MV complex, the commissural leaflets have the ability to fully flex to the annular level, moving perpendicularly to the AMVL and posterior MVL (PMVL) due to its anatomical position; this provides maximal opening during diastole. In systole, the commissural leaflets help create a tight seal with neighboring leaflet segments (e.g., C1, A1, P1) from the summation of multiple coaptation sites. These movements are made possible because of the unique fan-shaped chordal insertion onto the leaflets.

**Fig. 2:**
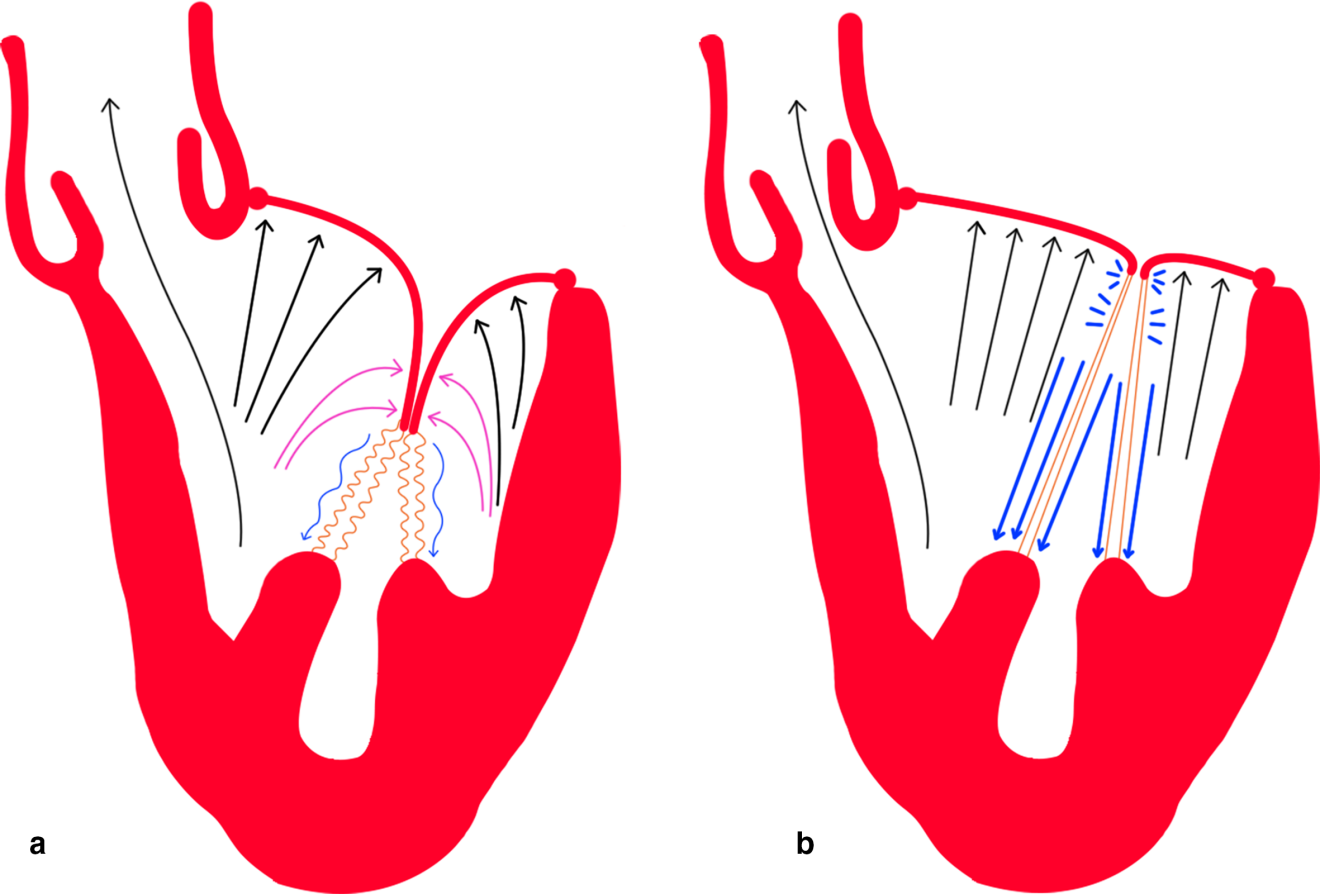
Systolic ventricular pressure distribution on mitral leaflets in (**a**) dipped coaptation and (**b**) edge-to-edge coaptation. The black arrows show the ventricular pressure that both mitral leaflets must counter to prevent regurgitation, which is greater in (**b**). The pink arrows in (**a**) represent the equal and opposite forces against the anterior and posterior mitral leaflet edges that nullify the ventricular pressure at those points. Note the larger left ventricular outflow tract in (**a**) with dipped coaptation, decreasing resistance to systolic flow and reducing the risk of systolic anterior motion. The blue arrows represent chordal tension, differing in thickness to represent the exertional force on the chords (colored orange). Note the chords are depicted ‘wavily’ in (**a**) to emphasize reduced chordal tension.

**Fig. 3:**
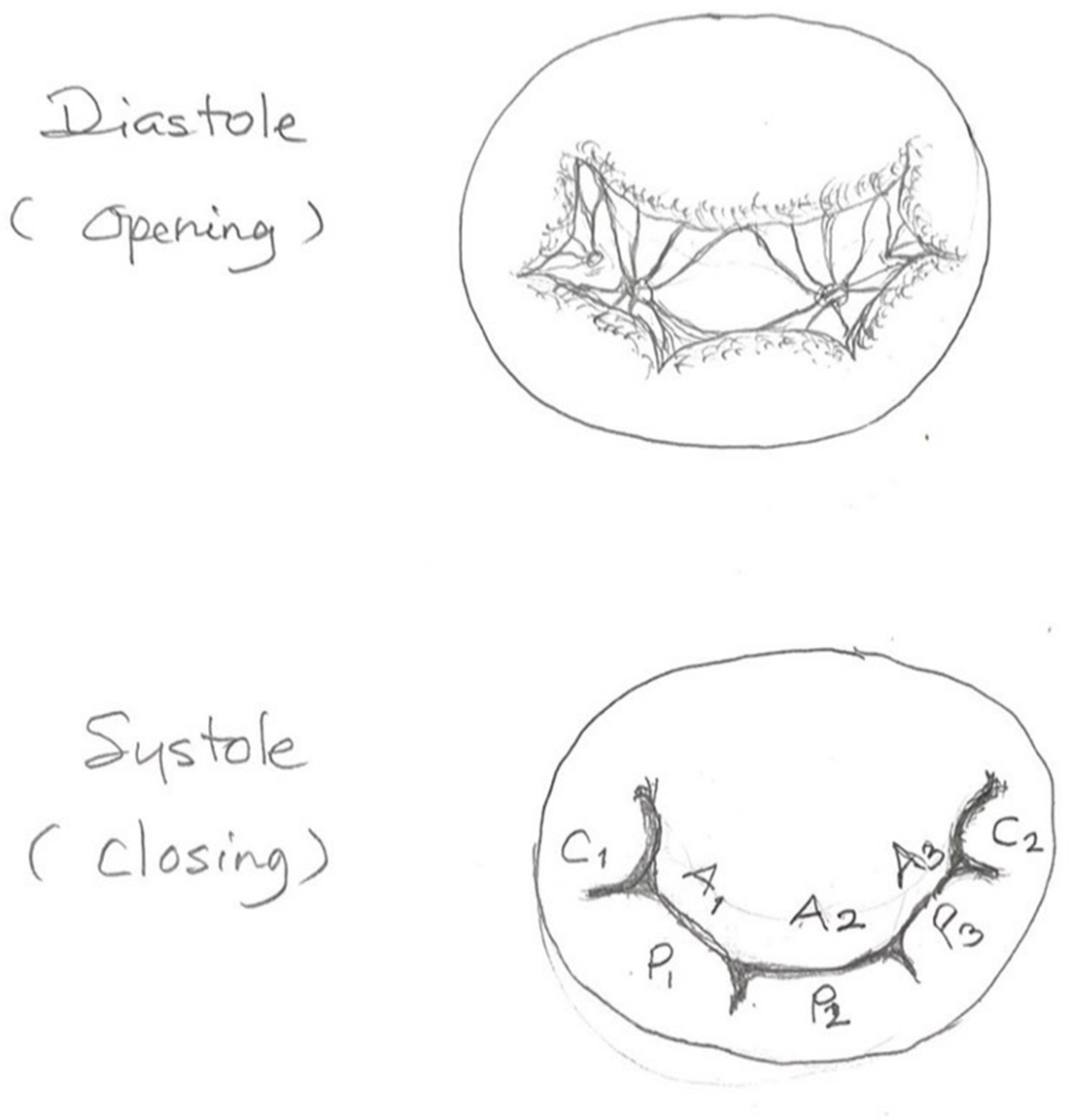
Dynamics of the mitral valve complex during diastole and systole, including the commissural leaflets.

If valve closure was edge-to-edge, the AMVL and PMVL would experience much greater force; this would demand tremendous tension in the chords, particularly the marginal ones, to prevent MR (Fig. 2b). However, dipped coaptation reduces chordal stress (Fig. 2a) because there is greater distribution of tension across more orders of chordae.^21^ Much like the architecture of an arch, the commissural leaflets act like springers to support the keystone of coaptation. Thus, the commissural leaflets have a vital role in creating this design that exerts as little stress as possible on the leaflets and chords during systole, extending durability, and maximizing orifice opening during diastole. Figure 3 illustrates the MV dynamics during the cardiac cycle, including the commissural leaflets’ involvement. However, these benefits are only optimized in rigid leaflets if repair is further compounded by leaflet techniques, including peeling, to increase pliability.

#### Leaflet peeling and shaving

**Fig. 4:**
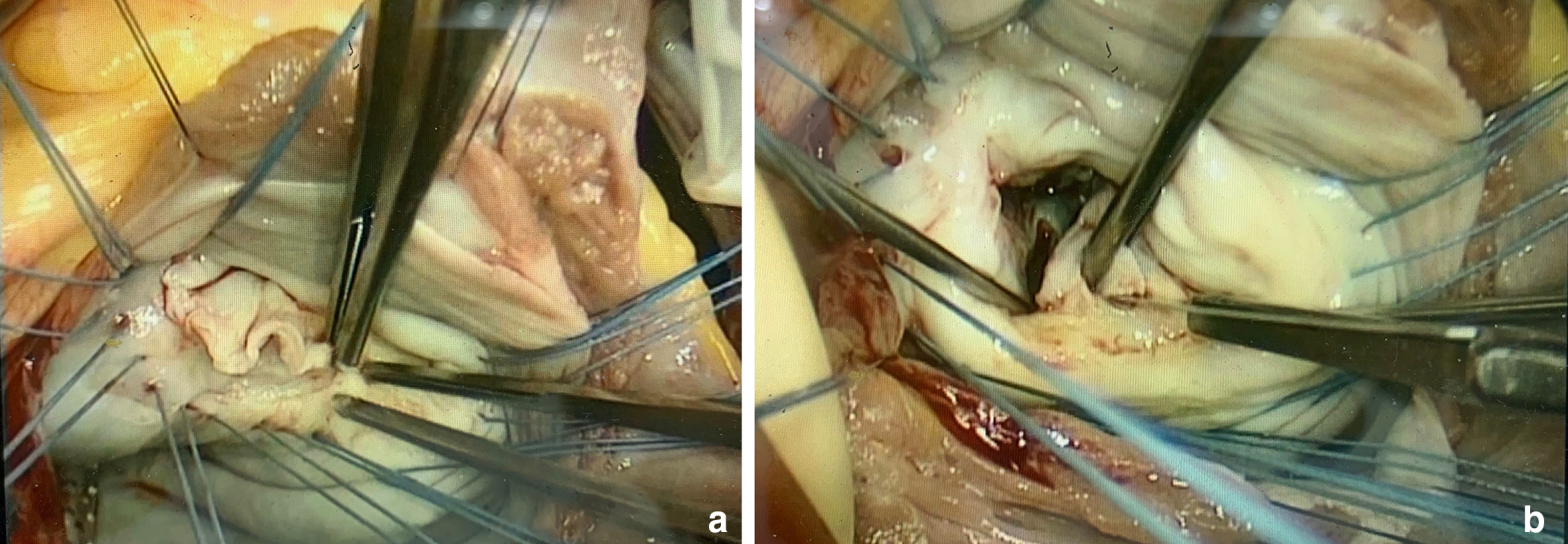
(**a**) Leaflet peeling; (**b**) posterior mitral leaflet fenestration and chordal resection.

Kumar’s group^22^ introduced the technique of leaflet thinning to help restore pliability of fibrotic valves,^22,23^ and has since been popularized by Chotivatanapong.^11^ Leaflet peeling and shaving helps achieve good leaflet coaptation by directly resecting diseased tissue, representing an integral technique in the rheumatic MVr toolbox (Fig. 4a).^4,11,24^ Beginning from the hinge, blunt dissection is used to gently peel off the fibrous layer covering the atrial leaflet surface, stopping at the rough zone. Calcifications in the rough zone, or more “sticky” areas, should be shaved off as much as possible using sharp dissection. Any leaflet perforation can be fixed by simple sutures. In our experience, finding a good plane is key in successful peeling and preventing fibrous ridges; understanding of the histological layers help justify this approach. The leaflet body, or the smooth zone, predominantly consists of the atrialis layer which contains well-aligned collagen and elastin sheets.^19^ Conversely, since the major tissue layer of the free edge is the spongiosa, the more loosely organized collagen makes it more difficult to find a homogenous plane.^19^ Therefore, leaflet peeling is extremely useful in the smooth zone, whilst leaflet shaving is reserved for the rough zone.

Limitations of leaflet peeling include incomplete fibrous tissue removal and difficulty performing it on the ventricular side due to both impracticalities of exposure and the irregular surface containing chordal attachments. However, the benefits of restoring even just acceptable rather than full mobility significantly outweighs the technical drawbacks. As mentioned previously, dipped coaptation is integral in normal MV dynamics, heavily implicated in the modified techniques of leaflet extension or replacement that several authors utilize to maximize surface area for greater coaptation.^4,18^

Apart from augmenting the coaptation surface and thus restoring the compensatory mechanism in the event of annular dilatation, the restored pliability serves as a protective mechanism during systole. Leaflets are normally expandable, physiologically important in distributing stress during systole by expansion of the leaflet tenting area, reducing chordal tension and extending durability as mentioned previously. In diastole, pliability not only enables maximal opening of the MV orifice, but the leaflet free edge curvature itself helps guide diastolic blood flow towards the apex. Thus, leaflet peeling is a brilliant technique to directly address rheumatic lesions and the fundamental objective of leaflet pliability.

### Chordae Tendineae

Thickened chords, either shortened or elongated, and chordal fusion, are very common findings in RHD,^11^ with the secondary chordae often responsible for valve thickening. This causes obstruction in diastole, and restriction in systole. Two chordal techniques addressing mainly patients with MS will be discussed (Fig. 4b).

#### Chordal resection

To fix chordal thickening, simple resection of the responsible chord is useful, but often limited to only secondary or tertiary chordae.^16^ However, resection can be performed on all orders of chordae.^11,15^

In our experience, chordal repair is always indicated when the primary chord of the AMVL is resected; chordal transfer or neochordal implantation is performed for anticipatory prolapse upon marginal chordae resection, important in preventing MR which is commonly seen in younger patients if left unrepaired. This is due to the longer linear coaptation-to-hinge distance of the AMVL producing greater tension, hence requiring chordal repair. Conversely, chordal repair can go unreplaced in certain cases after PMVL primary chord resection as the same is not true in the PMVL. Additionally, in our experience, the fibrotic RHD tissue on the PMVL ventricular side can be paradoxically advantageous in foregoing chordal implantation or transfer post-resection because the tough fibrotic material itself lessens the likelihood of MR to occur.

#### Chordal fenestration

Fenestration is a widely employed technique in which a triangular wedge is cut from the fused chord^16^ and marked down for papillotomy. This is important because even if the MV orifice is sufficiently open, diastolic obstruction can still occur if thickened chords remain. Restoring multiple channels between chords will reduce resistance to restore collateral flow during diastole, which will also accommodate circumstances demanding increased cardiac output. Furthermore, fenestration allows increased transverse mobility of leaflets during systole to better distribute tension. Thus, this technique helps alleviate leaflet restriction and release subvalvular stenosis.

### Annulus

The mitral annulus is a pliable, fibrous ring acting as the anatomical junction between the left atrium, LV, and leaflets.^19,20^ The unique nonplanar saddle shape geometry confers mechanical advantage, dynamically varying during the cardiac cycle. During diastole, the annulus displays less curvature and is more circular in shape, allowing formation of a greater MV orifice to maximize blood flow into the LV. In systole, the annulus displays greater curvature, changing into the prominent saddle-shape maximizing leaflet coaptation due to a smaller orifice and thus MV closure, as well as widening the left ventricular outflow tract due to the aorto-mitral fibrous continuity.^19^ Mitral annular size, shape, and dynamics may vary substantially across rheumatic populations; dilatation, fibrosis, and deformity could all occur together. Hence, mitral annuloplasty using a prosthetic ring is a mandatory procedure in almost all RHD cases, regardless of excellent leaflet repair, as mitral dynamics and durability would remain unsatisfactory without an adequate annulus.^7,10,11,15^

The ring holds many roles, besides providing a framework for the MV complex, which include restoring effective dynamic compliance during both diastole and systole, enhancing MV opening and closure, maximizing coaptation, minimizing chordal stress, and preventing further dilatation. Careful ring sizing is mandatory to stabilize MVr with optimal MV opening and durable valve competency. In our experience, valve sizing techniques by measuring the AMVL tissue advocated by Carpentier is a very useful guideline. However, if there is doubt despite careful sizing, a smaller ring is chosen. Due to the retracted valve tissue from the rheumatic inflammatory process, a smaller ring would provide better closure and coaptation in this situation. Additionally, the greater the annular deformity, the more preferable a rigid ring is in order to stabilize and correct annular geometry, as a flexible ring would simply accommodate the rheumatic deformity, defeating the aim of restoring normal annular dynamics.

## Future Directions

The era of rheumatic MVr surpasses that of the long-standing “repair versus replacement” debate. While reoperation remains a potent matter, it is now a conundrum of extending repair to more complicated pathology usually portending an almost certain favor of MVR, including mixed lesions, calcified MS, moderate-to-severe MR, and severe calcification and fibrosis of the leaflets or subvalvular apparatus.^3,18,25^ The “*French Correction*”^16^ techniques remain extensively employed today, but struggle to independently address these difficult rheumatic lesions. However, repair has become more feasible, safe, and reproducible with improved understanding, reducing previous limitations.^11,26^

Indeed, the current developmental trend of “nonclassical” techniques emphasize resection of fibrous tissue plaguing valves. According to several authors, this strategy may lower surgeon reluctance to repair and enhance durability.^3,11,15,18,26^ The phenomenon of post-repair MV failure due to residual diseased tissue was first reported in the Chauvaud et al. long-term series,^12^ and another study found recurrent inflammatory damage to be the main cause of mid-term reoperation peak.^23^ This could help explain why Choudhary et al.^24^ identified usage of cuspal thinning as an important predictor in developing MR post-repair in an earlier study; since Kumar’s group pioneered this now widely-employed technique,^22^ it is unlikely cuspal thinning directly caused inferior durability in this series. Instead, usage of the technique is likely a surrogate marker for more complicated rheumatic lesions that possess a higher probability of residual diseased tissue post-repair. Thus, adopting an aggressive resection approach to lower the risk of recurrence and/or progression of RHD while preserving as much native tissue as possible is a promising strategy. Focusing on “nonclassical” tactics has been shown to obtain exceptional durability (10-year freedom from reoperation, 94% ± 5%).^26^ Furthermore, despite similarly reporting leaflet-related techniques to be a significant risk factor of valve failure like Choudhary et al.,^24^ Dillon et al.^10^ not only mentioned reserving such methods for severe RHD but adopted an aggressive approach using novel techniques with ultimately excellent clinical outcomes. This leads us to the complicated issue of surgical timing and patient selection. Although no clear consensus exists, it is a reminder that rheumatic populations are diverse, partially explaining the relatively inconsistent findings in the literature versus degenerative disease. As previously mentioned, active inflammation at surgery is a significant predictor of inferior outcome;^2,17^ this in turn has an association with younger patients in endemic developing regions with greater susceptibility to relapsing inflammation. One may infer that this could explain the superior results in studies involving only “burnt-out” rheumatics,^10^ but a clinical team’s superb skills and experience must not be forgotten. Improved understanding of the MV complex-dynamic interplay and unique challenges RHD poses have reduced repair limitations. As this field matures, an “aggressive” approach is duly required to expand both the surgical and mental boundaries.

## Conclusions

We firmly believe that MVr can be offered to nearly all RHD patients with brilliant outcomes. Ultimately, techniques must be tailored to serve the fundamental principle of achieving good coaptation concomitant with a synergistic understanding of the cardiac cycle and rheumatic pathophysiology. In turn, more calculated decisions will be made when selecting the most suitable repair techniques aimed at enhancing durability; this critical shift in mindset must be adopted in rheumatic MVr. With great appreciation and respect of nature’s perfectly balanced design of the MV complex, repair will endure.

## Supplemental Material

Table S1 - Supplemental material for Current Perspectives on Contemporary Rheumatic Mitral Valve Repair Click here for additional data file. Supplemental material, Table S1, for Current Perspectives on Contemporary Rheumatic Mitral Valve Repair by Chaninda Dejsupa, Taweesak Chotivatanapong, Massimo Caputo and Hunaid A. Vohra in Innovations: Technology and Techniques in Cardiothoracic and Vascular Surgery

Visual Abstract - Supplemental material for Current Perspectives on Contemporary Rheumatic Mitral Valve Repair Click here for additional data file. Supplemental material, Visual Abstract, for Current Perspectives on Contemporary Rheumatic Mitral Valve Repair by Chaninda Dejsupa, Taweesak Chotivatanapong, Massimo Caputo and Hunaid A. Vohra in Innovations: Technology and Techniques in Cardiothoracic and Vascular Surgery
