# Supplementary material for: Current Perspectives on Contemporary Rheumatic Mitral Valve Repair
Source: Innovations (Phila). 2021 Sep 3;16(6):510–6. doi: 10.1177/15569845211032942 (PMC8679173; doi:10.1177/15569845211032942)
Supplement: Table S1 - Supplemental material for Current Perspectives on Contemporary Rheumatic Mitral Valve Repair [file sj-docx-1-inv-10.1177_15569845211032942.docx]

**Supplemental Table:** Summary of Rheumatic Mitral Valve Repair Series

| **Study** | **Design** | **Country** | **Study year** | **Study period** | **Sample size** | **Repair rate** | **Mean follow-up, mons** | **Freedom from reoperation** | **Freedom from valve-related event** ^a^ | **Freedom from mitral dysfunction** |
| --- | --- | --- | --- | --- | --- | --- | --- | --- | --- | --- |
| Chotivatanapong^1^ | Retrospective observational | Thailand | 2020 | 2003-2016 | 534 | NR | 41.9 | 12 patients | 57 readmissions | NR |
| Waikittipong^2^ | Retrospective observational | Thailand | 2015 | 2003-2014 | 97 | NR | 58.8±4.2 | 82.7% (10 yrs) | 56.4% (10 yrs) | NR |
| Fu et al.^3^ | Prospective observational | China | 2020 | 2011-2019 | 612 | 37.2% | 49.4 | >85% (8 yrs) | >95% (8 yrs) | Freedom from >1+ MR: >93% (11 yrs) |
| Dillon et al.^4^ | Prospective observational | Malaysia | 2014 | 1997-2011 | 253 | 40.6% (34.5% in >55 yrs of age) | 47.8 | 98.4% (10 yrs) | 81.6% (10 yrs) | Freedom from >1+: 89.5% (at last follow-up) |
| El Oumeiri et al.^5^ | Retrospective observational | Belgium | 2009 | 1996-2007 | 78 | 59.1% | 52±37 | 94% (10 yrs) | 86% (10 yrs) | Freedom from >2+ MR: 83% (8 yrs) |
| Kumar et al.^6^ | Retrospective observational | India | 2006 | 1988-2003 | 898 | 37.6% | 62.7±31.8 | 81% (10 yrs) | 32% (10 yrs) | Freedom from >1+ MR: 32% (10 yrs) |
| Choudhary et al.^7^ | Retrospective observational | India | 2001 | 1988-1999 | 818 (718 RHD) | NR | 44.9±33.2 | 65.2% (11 yrs) | 38.1% (11 yrs) | Freedom from >1+ MR: 52.3% (11 yrs) |
| Chauvaud et al.^8^ | Retrospective observational | France | 2001 | 1970-1994 | 951 | NR | 147.6 | 82% (10 yrs), 55% (20 yrs) | 82%, 72%, 52% (10, 15, & 20 yrs) | NR |
| Pomerantzeff et al.^9^ | Retrospective observational | Brazil | 2009 | 1985-2005 | 330 | NR | <240 | 30.4% (20 yrs) | 95.6% (endocarditis-free), 99.7% (thromboembolism-free) | NR |
| Yau et al.^10^ | Prospective observational | Canada | 2000 | 1978-1995 | 142 | 24.8% | 78±4 | 72% (10 yrs) | 71% (10 yrs) | NR |
| DiBardino et al.^11^ | Retrospective observational | America | 2009 | 1972-2008 | 193 | NR | 360 | 66% (10 yrs), 34% (20 yrs), 10% (30 yrs) | NR | NR |
| Kim et al.^12^ | Retrospective observational | Korea | 2012 | 1997-2010 | 193 | NR | 76.7±45.6 | 96.7% (10 yrs) | 85.5% (10 yrs) | Freedom from moderate to severe mitral dysfunction: 66.4% (10 yrs) |
| Kim et al.^13^ | Retrospective observational | Korea | 2017 | 1997-2015 | 294 | 16.9% | 130.9±27.7 | 81.9% (15 yrs) | NR | Freedom from moderate to severe mitral dysfunction: 68.0% (10 yrs) |
| Moorthy et al.^14^ | Retrospective observational | Malaysia | 2019 | 1992-2015 | 336 | 80.2% | 67.2 | 81.7% (10 yrs) | 87.9% (10 yrs), 82.9% (20 yrs) | NR |
| McGurty et al.^15^ | Retrospective observational | Australia | 2019 | 1997-2015 | 79 | NR | 92.64 | 53% (10 yrs), 49% (15 yrs) | NR | Freedom from mitral deterioration (late MV reoperation or greater than moderate MR or MS):  36% (10 yrs), 28% (15 yrs) |
| Remenyi et al.^16^ | Retrospective observational | New Zealand | 2013 | 1990-2006 | 81 | 65.4% | 91.2 | 76% (10 yrs), 76% (14 yrs) | 67% (10 yrs),  67% (14 yrs) | NR |

Abbreviations: IE, infective endocarditis; MR, mitral regurgitation; NR, not reported; RHD, rheumatic heart disease.

^a^Definition of valve-related events:

- Late death, reoperation, MR progression (Waikittipong ^2^)
- Neurologic thromboembolism, bleeding events, IE (Fu et al. ^3^)
- Valve failure: recurrent significant MR of >1+ MR or mitral valve reoperation; valve-related complications (no patients experienced first three): thromboembolism, IE, bleeding secondary to anticoagulation, reoperation (Dillon et al. ^4^)
- Valve-related death, reoperation, IE, thromboembolism (El Oumeiri et al. ^5^)
- “Guidelines for Reporting Morbidity and Mortality After Cardiac Valvular Operations,” 1996 (Kumar et al. ^6^)
- Late death, reoperation, thromboembolism, IE, hemolysis (Choudhary et al. ^7^)
- Hospital death, reoperation, late death, thromboembolism (Chauvaud et al. ^8^)
- Endocarditis-free, thromboembolism-free (Pomerantzeff et al. ^9^)
- Thromboembolism, IE, reoperation (Yau et al. ^10^)
- “Guidelines for Reporting Morbidity and Mortality After Cardiac Valvular Operations,” 2008 (Kim et al. ^12^)
- “Ad Hoc Liaison Committee for Standardizing Definitions for Prosthetic Heart Valve Morbidity,” 2008 (Moorthy et al. ^14^)
- Late death, thrombosis, embolism, hemorrhage, endocarditis, and reoperation (Remenyi et al. ^16^)

**References**

1. Chotivatanapong T. Rheumatic mitral valve repair: a personal perspective and results. *Asian Cardiovasc Thorac Ann* 2020; 28: 366-370.

2. Waikittipong S. Mitral valve repair for rheumatic mitral regurgitation: mid-term results. *Asian Cardiovasc Thorac An* 2015; 23: 658-664.

3. Fu J, Li Y, Zhang H, et al. Outcomes of mitral valve repair compared with replacement for patients with rheumatic heart disease. *J Thorac Cardiovasc Surg* 2021; 162: 72-82.

4. Dillon J, Yakub MA, Kong PK, et al. Comparative long-term results of mitral valve repair in adults with chronic rheumatic disease and degenerative disease: is repair for "burnt-out" rheumatic disease still inferior to repair for degenerative disease in the current era? *J Thorac Cardiovasc Surg* 2015; 149: 771-779.

5. El Oumeiri B, Boodhwani M, Glineur D, et al. Extending the scope of mitral valve repair in rheumatic disease. *Ann Thorac Surg* 2009; 87: 1735-1740.

6. Kumar AS, Talwar S, Saxena A, et al. Results of mitral valve repair in rheumatic mitral regurgitation. *Interact Cardiovasc Thorac Surg* 2006; 5: 356-361.

7. Choudhary SK, Talwar S, Dubey B, et al. Mitral valve repair in a predominantly rheumatic population. Long-term results. *Tex Heart Inst J* 2001; 28: 8-15.

8. Chauvaud S, Fuzellier J-F, Berrebi A, et al. Long-term (29 years) results of reconstructive surgery in rheumatic mitral valve insufficiency. *Circulation* 2001; 104(suppl_1): I12-I15.

9. Pomerantzeff P, Brandão C, Filho O, et al. Mitral valve repair in rheumatic patients with mitral insuficiency: twenty years of techniques and results. *Rev Bras Cir Cardiovasc* 2009; 24: 485-489.

10. Yau TM, El-Ghoneimi YAF, Armstrong S, et al. Mitral valve repair and replacement for rheumatic disease. *J Thorac Cardiovasc Surg* 2000; 119: 53-61.

11. DiBardino DJ, ElBardissi AW, McClure RS, et al. Four decades of experience with mitral valve repair: analysis of differential indications, technical evolution, and long-term outcome. *J Thorac Cardiovasc Surg* 2010; 139: 76-84.

12. Kim GS, Lee CH, Kim JB, et al. Echocardiographic evaluation of mitral durability following valve repair in rheumatic mitral valve disease: impact of Maze procedure. *J Thorac Cardiovasc Surg* 2014; 147: 247-253.

13. Kim WK, Kim HJ, Kim JB, et al. Clinical outcomes in 1731 patients undergoing mitral valve surgery for rheumatic valve disease. *Heart* 2018; 104: 841-848.

14. Krishna Moorthy PS, Sivalingam S, Dillon J, et al. Is it worth repairing rheumatic mitral valve disease in children? Long-term outcomes of an aggressive approach to rheumatic mitral valve repair compared to replacement in young patients. *Interact Cardiovasc Thorac Surg* 2019; 28: 191-198.

15. McGurty D, Remenyi B, Cheung M, et al. Outcomes after rheumatic mitral valve repair in children. *Ann Thorac Surg* 2019; 108: 792-797.

16. Remenyi B, Webb R, Gentles T, et al. Improved long-term survival for rheumatic mitral valve repair compared to replacement in the young. *World J Pediatr Congenit Heart Surg* 2013; 4: 155-164.
