# Supplementary material for: Current Perspectives on Contemporary Rheumatic Mitral Valve Repair
Source: Innovations (Phila). 2021 Sep 3;16(6):510–6. doi: 10.1177/15569845211032942 (PMC8679173; doi:10.1177/15569845211032942)
Supplement: Visual Abstract - Supplemental material for Current Perspectives on Contemporary Rheumatic Mitral Valve Repair [file sj-pptx-2-inv-10.1177_15569845211032942.pptx]

## Slide 1
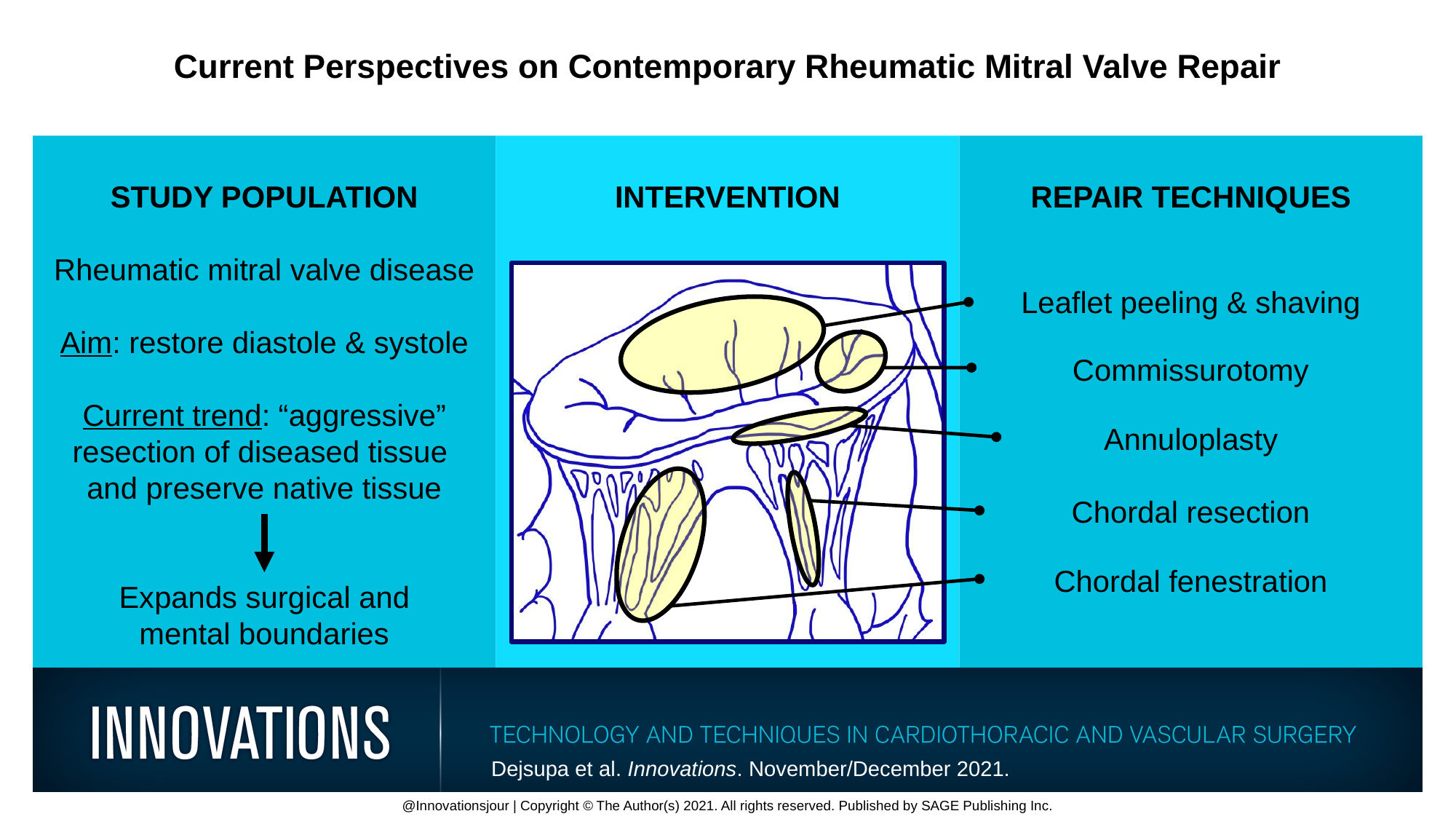

# Current Perspectives on Contemporary Rheumatic Mitral Valve Repair
STUDY POPULATION
Rheumatic mitral valve disease
Aim: restore diastole & systole
Current trend: “aggressive” resection of diseased tissue
and preserve native tissue
Expands surgical and
mental boundaries
INTERVENTION
REPAIR TECHNIQUES
Leaflet peeling & shaving
Commissurotomy
Annuloplasty
Chordal resection
Chordal fenestration
Dejsupa et al. Innovations. November/December 2021.
@Innovationsjour | Copyright © The Author(s) 2021. All rights reserved. Published by SAGE Publishing Inc.
